# Towards Robust Decision-Making for Autonomous Highway Driving Based on Safe Reinforcement Learning

**DOI:** 10.3390/s24134140

**Published:** 2024-06-26

**Authors:** Rui Zhao, Ziguo Chen, Yuze Fan, Yun Li, Fei Gao

**Affiliations:** 1 College of Automotive Engineering, Jilin University, Changchun 130025, China; rzhao@jlu.edu.cn (R.Z.); yangjy1520@jlu.edu.cn (Z.C.); fanyz23@mails.jlu.edu.cn (Y.F.); 2 Graduate School of Information and Science Technology, The University of Tokyo, Tokyo 113-8654, Japan; li-yun@g.ecc.u-tokyo.ac.jp; 3 State Key Laboratory of Automotive Simulation and Control, Jilin University, Changhun 130025, China

**Keywords:** autonomous driving, importance sampling, catastrophic forgetting, deep reinforcement learning, constrained policy optimization

## Abstract

Reinforcement Learning (RL) methods are regarded as effective for designing autonomous driving policies. However, even when RL policies are trained to convergence, ensuring their robust safety remains a challenge, particularly in long-tail data. Therefore, decision-making based on RL must adequately consider potential variations in data distribution. This paper presents a framework for highway autonomous driving decisions that prioritizes both safety and robustness. Utilizing the proposed Replay Buffer Constrained Policy Optimization (RECPO) method, this framework updates RL strategies to maximize rewards while ensuring that the policies always remain within safety constraints. We incorporate importance sampling techniques to collect and store data in a Replay buffer during agent operation, allowing the reutilization of data from old policies for training new policy models, thus mitigating potential catastrophic forgetting. Additionally, we transform the highway autonomous driving decision problem into a Constrained Markov Decision Process (CMDP) and apply our proposed RECPO for training, optimizing highway driving policies. Finally, we deploy our method in the CARLA simulation environment and compare its performance in typical highway scenarios against traditional CPO, current advanced strategies based on Deep Deterministic Policy Gradient (DDPG), and IDM + MOBIL (Intelligent Driver Model and the model for minimizing overall braking induced by lane changes). The results show that our framework significantly enhances model convergence speed, safety, and decision-making stability, achieving a zero-collision rate in highway autonomous driving.

## 1. Introduction

Autonomous vehicles (AVs) have the potential to revolutionize transportation systems, and have attracted great attention in recent research and development. However, despite the excellent performance of autonomous driving products showcased by companies such as Waymo and Baidu Apollo in normal traffic, their safety reports document many emergency takeover incidents that occur when facing unknown or complex situations, such as visual barriers, unsignalized intersections, and multi-intersection sections. Designing robust and safe autonomous driving decisions remains a challenge. Although with the development of technology, vehicle road collaborative perception technology has shown the potential to improve vehicle perception accuracy, reduce latency, and eliminate perception blind spots by integrating information from various sources, which is expected to improve the safety and accuracy of autonomous driving decisions. And relevant research has proven this. Studies have shown that collaborative perception, enabled by wireless communication technology, facilitates the integration of environmental data from edge nodes with local sensory information, thereby enhancing vehicle operation efficiencies [1]. Further investigations have revealed that interconnected vehicles can surpass physical barriers in perception through cooperative strategies, substantially boosting safety across various traffic conditions [2]. Additionally, the critical role of vehicle-to-everything (V2X) communication in collective perception has been underscored. This technology not only refines the accuracy and completeness of environmental perception around vehicles but also stresses the essential market penetration of V2X services to markedly enhance traffic safety [3]. However, making accurate, safe, and robust decisions based on perceptual information is still a challenge for AVs, and it is also the foundation of full modal autonomous driving.

Operating on highways, AVs represent a technology that aims to provide safer, more efficient, and environmentally friendly transportation. The complexity of the highway environment, characterized by high-speed maneuvers, dense traffic, and unexpected situations, requires robust decision-making policies. Traditional autonomous driving methods are typically divided into rule-based and optimization-based approaches. Rule-based methods rely on predefined explicit instructions to handle scenarios and make decisions. However, they often lack adaptability. The predefined rules usually fail to cover all possible driving situations, especially in complex and unpredictable environments. Additionally, rule systems often depend on the designers’ understanding of the problem, which limits the potential for innovation and optimization of solutions [4,5]. Optimization-based methods use mathematical optimization to search and compute within a vast solution space, aiming to maximize objective values and determine the optimal control strategy [6,7]. However, these methods have low computational efficiency and usually require several seconds to resolve issues. They demand high computational performance from the control units, making it challenging to meet the millisecond-level real-time requirements of control outputs in autonomous driving applications [8].

Reinforcement Learning (RL) methods, particularly deep RL, have recently emerged as a powerful learning framework for mastering autonomous driving decisions in complex traffic environments. RL-based decision-making methods possess the capability for self-evolution through exploration, enabling AVs to learn optimal behaviors through interaction with the environment. These methods hold the potential to address challenges such as suboptimality and computational inefficiency, which are encountered by current rule-based and optimization-based approaches. Thus, RL has become a key technology for enabling advanced automation in AVs. Its ability to adjust and optimize decision-making processes in real-time positions it at the forefront of autonomous driving policy development for AVs [9].

The development of RL in the field of autonomous driving has transitioned from foundational models to advanced algorithms capable of addressing complex and dynamic driving tasks. Early RL methods focused on simple control tasks[10], laying the foundation for more complex methods such as Deep Q-Network (DQN) for higher-dimensional state and action spaces [11], Deterministic Policy Gradient (DDPG) [12,13], Proximal Policy Optimization (PPO) [14], Trust Region Policy Optimization (TRPO) [14], and Asynchronous Advantage Actor Critic (A3C) [15], among others [16]. These methods have been used in the field of autonomous driving and have demonstrated good performance [15,17,18]. However, despite the remarkable performance of autonomous driving products showcased by companies such as Waymo, Baidu Apollo, and others in regular traffic, their safety reports have documented numerous emergency takeover incidents when faced with unknown or complex situations. Concurrently, recent research indicates that public acceptance of AVs is primarily concerned with safety [19,20]. The trial-and-error nature of RL facilitates policy evolution, but an optimal policy without constraint guarantees poses risks to autonomous vehicle driving safety. Safety constraints must be aligned with RL, such as by sampling dangerous states and reformulating optimization criteria [21,22,23]. Therefore, it is essential to investigate additional safety guarantees to bridge the gap between the forthcoming autonomous future and the current state-of-the-art technologies [24,25,26].

The Constrained Markov Decision Process (CMDP) is a key framework for safe RL, particularly for tasks where safety constraints are non-negotiable, such as autonomous driving or human-interactive robotics [27]. CMDPs extend the standard MDP by incorporating constraints on actions or states, ensuring that the learning process adheres to safety constraints and thus prevents hazardous behavior during training and deployment stages [27,28]. The Constrained Policy Optimization (CPO) algorithm aims to address CMDPs by optimizing policies within constraints [27]. It has brought revolutionary advancements to safe RL, specifically designed to integrate safety constraints into the RL optimization process. This type of policy optimization ensures that the learning process seeks to maximize rewards while adhering to predefined safety constraints, making it particularly suitable for safety-critical applications such as autonomous driving. The emergence of the CPO algorithm provides a theoretical basis for this type of policy optimization, namely, the security reinforcement learning algorithm [27,29]. However, while the traditional CPO framework is crucial for managing risks in safe RL by facilitating policy updates that simultaneously achieve reward optimization and constraint satisfaction, its dependence on online learning introduces significant limitations, particularly in effectively utilizing past experiences. This limitation stems from the continually evolving nature of policy models. With updates to the models, past data may become less relevant or actionable, potentially leading to inefficient learning and adaptation, or even catastrophic forgetting. Such challenges make it difficult to learn safe, robust, and well-generalized driving policies [30,31].

To address the aforementioned limitations, this paper introduces Replay Buffer Constrained Policy Optimization (RECPO), an innovative algorithm that advances traditional CPO. RECPO incorporates an experience replay pool that utilizes importance sampling, a crucial innovation allowing for the adjustment of the utility of past experiences to align with the current policy model. This innovation tackles the challenges of low data efficiency and underutilization of historical learning. Additionally, RECPO maintains the safety assurances provided by CPO, thereby significantly enhancing learning efficiency, exploration capabilities, and robustness in dynamic environments and against rare events. We have applied RECPO to the learning of autonomous driving decisions on highways with a focus on improving safety, efficiency, and comfort. Furthermore, by enabling the algorithm to adaptively reuse past experiences, RECPO effectively addresses common issues such as catastrophic forgetting and long-tail effects in autonomous driving scenarios.

The main contributions of this work are as follows:

(1) The decision-making problem for highway autonomous driving is modeled as a safe reinforcement learning formulation, CMDP. This formulation includes a well-designed state and action space, as well as reward and safety cost functions. We introduce the RECPO algorithm to solve CMDP, which incorporates an independent cost neural network specifically designed to constrain risky behaviors within the agent system, ensuring that solutions remain within safe limits. This approach successfully overcomes the limitations of traditional reinforcement learning methods in safety-critical fields such as autonomous driving, maintaining safety while seeking performance optimization. After training and deployment, the strategy demonstrated robust performance.

(2) The proposed RECPO algorithm introduces a specialized training data storage mechanism that collects and retains data during agent operations. During the training phase, the importance sampling based on the probability distribution of policy updates allows for the reuse of historical data, effectively mitigating catastrophic forgetting and long-tail distribution issues, thereby enhancing the efficiency and robustness of the learning process.

(3) The proposed RECPO-based decision-making algorithm was extensively tested in typical highway scenarios, and the experimental results showed that compared to traditional CPO, DDPG, and IDM + MOBIL (Intelligent Driver Model and the model for minimizing overall braking induced by lane changes), RECPO exhibited faster convergence, higher safety, and decision stability.

The remainder of this paper is organized as follows: Section 2 discusses related work, Section 3 first defines the problem and then presents the framework of this study, Section 4 discusses how to transform the highway autonomous driving decision task into a CMDP safe RL formalization framework for solving it with RECPO, Section 5 provides a detailed introduction to the RECPO algorithm, including its components and updating process, Section 6 discusses the experimental setup, analyzes the experimental process, and compares the experimental results, and Section 7 summarizes the paper.

## 2. Related Works

### 2.1. Rule Based and Optimization Computation Based for Autonomous Driving Decision Planning

In the field of autonomous driving, traditional decision-making and planning algorithms based on rules and optimization have been extensively studied and implemented. For instance, Wei et al. [4] developed rule-based optimal control strategies for autonomous vehicles that adhere to complex specifications imposed by traffic laws and cultural expectations, utilizing Control Lyapunov Functions (CLF) and Control Barrier Functions (CBF) to ensure safety and stability within the system. Bae et al. [32] provided a vehicle system framework based on Finite State Machines (FSM) for decision-making in urban environments to enhance traffic safety and flow in practical applications. Fan et al. [33] detailed the EM motion planner used in Baidu’s Apollo system, which integrates rule-based and optimization methods to enhance the decision-making capabilities of autonomous vehicles. Additionally, Urmson et al. [34] refined these methods, developing more complex decision systems to cope with the driving challenges in urban environments. IDM and MOBIL also play a crucial role in autonomous driving strategies. Treiber and Kesting [35] provided a comprehensive theoretical foundation for these models and showcased their applications across various traffic scenarios. At the same time, Vanholme et al. [36] demonstrated how to integrate these models into complex autonomous driving systems to improve vehicle behavioral decisions. In terms of route planning and optimization computation for autonomous vehicles, Ferguson and Stentz [37] proposed an adaptive path planning technique suitable for unknown and semi-structured environments. Paden et al. [38] reviewed motion planning and control techniques for autonomous vehicles in urban environments, providing a systematic framework for autonomous vehicle control strategies, including graph search, sample-based methods, and optimization-based methods. Liu et al. [39] introduced an advanced path planning framework for autonomous vehicles using model predictive control (MPC), which provides significant insights into the application of MPC in improving the adaptability and decision-making capabilities of autonomous driving systems in varying driving conditions. Liu et al. [39] proposed a novel MPC framework that integrates vehicle dynamics, environmental constraints, and real-time trajectory optimization. This approach allows the autonomous vehicle to calculate the most efficient path while considering potential future obstacles and changes in the environment, thereby optimizing safety and efficiency. Thrun et al. [40] proposed a system using a hybrid approach of rule-based and optimization computation employed by the Stanley aircraft, which successfully completed the 2005 DARPA Grand Challenge. Although these traditional algorithms are effective, they often rely on fixed rules and model assumptions, as well as extensive computation, limiting their adaptability in the face of unknown or dynamically changing environments. Specifically, there is a significant change in data distribution, such as situations outside predefined rules, these methods may fail to maintain high safety and efficiency.

### 2.2. Machine-Learning-Based Methods for Autonomous Driving Decision Planning

Advances in autonomous driving technology have spurred extensive research into machine-learning-based approaches. In recent years, machine learning, particularly deep RL, has increasingly been applied to autonomous driving decision-making, demonstrating significant potential for computational efficiency and generalizability. Current research in RL-based autonomous driving methods primarily focuses on learning optimal strategies through interaction with the environment. For instance, methods based on Q-learning have been developed for intelligent highway overtaking, guiding vehicles to travel in appropriate lanes and generate acceleration commands. Additionally, some studies have proposed a decision-making framework based on DDPG to control vehicles effectively and safely in emergency situations [12,18]. Others have introduced a method based on DQN for determining lane choices and acceleration for autonomous vehicles [19]. Tang et al. [41] proposed a decision controller with continuous action space for highway driving scenes based on SAC. Dosovitskiy et al. [42] employed deep RL to train autonomous vehicles in virtual environments. Furthermore, Shalev-Shwartz et al. [43] proposed a model-based RL method to enhance the safety and efficiency of autonomous systems on highways. Although these methods perform well in simulated environments, they often face challenges when applied in the real world. For example, the safety-driving model proposed by Pan, Y et al. [44] relies on performance in specific scenarios, which may not cover all real-world situations. These models tend to lack sufficient robustness and adaptability when dealing with unseen circumstances or dynamically changing environments. Existing RL methods still have significant room for improvement in ensuring high safety and stability. Most current research has not adequately addressed the long-tail data issue, leading to models that are prone to failure in rare or exceptional circumstances. Moreover, these methods are susceptible to catastrophic forgetting during continual policy updates, affecting the continuity and effectiveness of the learning process.

### 2.3. Research on Safe Reinforcement Learning

Safe RL aims to ensure the safety of the learning process and policy execution by incorporating safety mechanisms within the reinforcement learning framework. Research in this field typically focuses on constructing RL policies that maintain high safety standards during both the training and deployment phases. For example, Kendall Gillies et al. [45] explored combining formal methods with safe reinforcement learning to design autonomous driving systems. Kai Yang et al. [9] proposed a new decision-making framework that ensures the safety lower bound by integrating traditional rule-based methods with modern machine learning techniques. Cao et al. [46] introduced a new reinforcement learning algorithm that considers model confidence during the learning and decision-making processes, opting for lower-risk decisions to ensure the safety of autonomous driving decisions. Tian R et al. [47] improved human–machine interaction safety by introducing a game-theoretic human model aware of confidence levels. However, these methods do not directly address how to handle specific safety constraints and may not always ensure policy safety while maximizing rewards. Therefore, in environments where dangerous states might be encountered, these methods may not guarantee absolute operational safety. In contrast, Altman et al. proposed a framework based on CMDP [22,23] that not only considers reward maximization but also introduces constraints on actions or policies, often related to safety or other mandatory rules. In the CMDP framework, policy optimization must consider both reward maximization and constraint satisfaction. The introduction of CMDPs provides a foundation for safe RL. For example, Joshua Achiam et al.’s CPO algorithm is specifically designed for handling constrained problems in RL, ensuring that the optimized policy adheres to a set of predefined safety or other types of constraints [26]. Lu Wen et al. [48] proposed Parallel Constrained Policy Optimization (PCPO), which uses synchronous parallel learners to explore different state spaces while ensuring safety, thereby accelerating learning and policy updates. Xu et al. [49] introduced a Constrained Penalty Q-learning (CPQ) algorithm that enforces constraints by penalizing the Q-function for violations, learning robust policies that outperform several baselines. Additionally, SaFormer [50] uses conditional sequence modeling to derive policies that meet constraints from a fixed dataset, showing adaptability to different constraint thresholds. However, despite these methods increasing safety assurances, they often lack efficiency, complexity, and adaptability in non-static environments, especially vulnerable in the face of long-tail data and catastrophic forgetting. In the context of autonomous driving, catastrophic forgetting implies that when the vehicle system learns new road conditions or driving scenarios, it might forget how to effectively deal with previously encountered situations. This not only reduces the system’s adaptability but also potentially increases the risk when driving in unfamiliar or changing environments. This paper proposes a robust decision framework integrated with safety mechanisms to address the complex and variable road conditions encountered in autonomous driving. By introducing importance sampling and optimized Replay buffer management, this study aims to overcome the limitations of traditional safe reinforcement learning methods, thereby enhancing the practical application safety and stability of autonomous driving systems.

## 3. Problem Statement and Method Framework

### 3.1. System Model and Problem Statement

**Figure 1:**
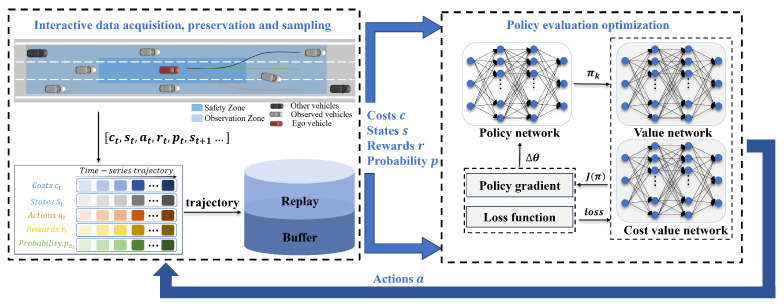
Schematic illustration of the proposed framework for robust decision-making in autonomous highway driving based on safe reinforcement learning.

The scenario considered in this study is illustrated in Figure 1, where the ego vehicle (EV) is on a typical multi-lane highway populated with surrounding vehicles (SVs) exhibiting random quantities and driving behaviors. The speeds of these SVs vary within the range $\left[v_{min},v_{max}\right]$, and they perform random yet reasonable lane changes. The observation area is defined as centered around the EV, extending to the outer lane lines on both sides and covering a distance of $d_{o}$ in both the front and rear directions. The EV can perceive the motion state information of the SVs within the observation area through either local sensors on the vehicle or connected sensors. Additionally, the safety zone is defined as centered around the EV, extending a distance of $d_{s}afe$ forward and backward, and stretching to the lane lines on both sides. This zone is identified as the critical safety area for driving.

**Figure 2:**
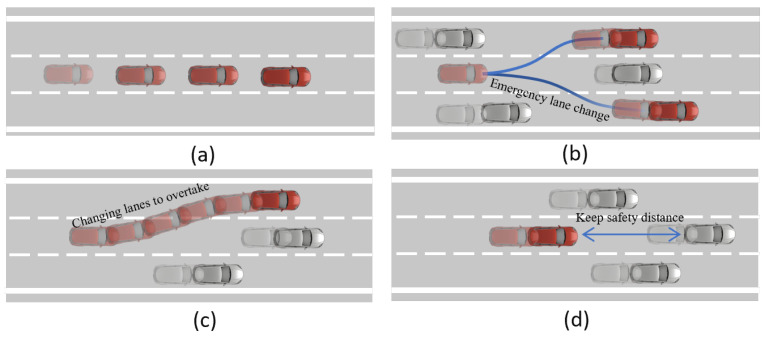
Four basic situations potentially encountered while driving on the highway. (**a**) Idle highways, changing lanes at will for acceleration and deceleration; (**b**) Emergency lane change to avoid the preceding vehicle; (**c**) Overtaking from the only available lane; (**d**) High speed roads with dense traffic flow can only slow down to follow.

Highway autonomous driving scenarios are abstracted into four categories, as shown in Figure 2: (a) In this scenario, vehicles on the highway are sparse, allowing the EV to change lanes and adjust speed within the limits allowed by traffic laws, providing a high degree of freedom for movement. Here, safety is almost unthreatened by external factors; (b) In this scenario, the EV encounters a vehicle in front that suddenly reduces speed, necessitating an urgent lane change to the left or right to avoid a collision; (c) In this scenario, there are vehicles in front of the EV as well as to the left, left–front, right, or right–front. The EV can only reduce speed to follow or overtake through the only lane that is clear of vehicles; (d) In this scenario, the highway is in a state of frequent use, with vehicles occupying all lanes ahead. The EV cannot overtake and must slow down and wait for an opportunity, representing the scenario with the highest risk factor. Real-world highways often involve a combination of two or more of these scenarios, along with vehicles’ random acceleration and lane changes, adding a high degree of unpredictability and complexity.

The state information *S* that the EV can observe at each time step includes a subset of its own vehicle state, a subset of surrounding vehicle states, and a subset of other environmental states:

$$S=(S_{EV},S_{SV},S_{EN})$$

where $S_{EV}=\left\{v_{e},\psi_{e}^{v},\psi_{e}^{r},x_{e},y_{e}\right\}$ represents the self-vehicle state subset, which includes the vehicle speed $v_{e}$ of the EV, the forward direction of EV $\psi_{e}^{v}$, the direction of the road $\psi_{e}^{r}$ and the coordinates of EV $\left(x_{e},y_{e}\right)$. $S_{SV}={\left\{x_{s}^{i},y_{s}^{i},v_{i}\right\}}_{i=1}^{8}$ represents a subset of surrounding vehicle states, which contains state information in eight directions. The nearest vehicle in each direction is considered the actual observation vehicle, and the observed data includes the coordinates $\left(x_{s}^{i},y_{s}^{i}\right)$ and speed $v_{i}$ of the vehicle. $S_{EN}=\left\{n_{ll},n_{rl}\right\}$ represents the environmental state space of the EV, including the number of left lanes $n_{ll}$ and the number of right lanes $n_{rl}$ where the EV is located.

The EV will output its own control information according to the state space at each time step, namely behavior $A=(a_{v},a_{c})$.

Therefore, the research problem is defined as follows: Obtain the limited information of the EV on surrounding traffic participants, road environment, and itself at each time step, and the highway autonomous driving system embedded with the RECPO policy will provide the EV’s control information for the next time step, including expected vehicle speed and driving direction, to ensure the safety, efficiency, and comfort of highway travel.

### 3.2. Framework for RECPO-Based Decision-Making in Autonomous Highway Driving

In the robust highway autonomous driving decision method proposed in this study based on RECPO, three neural networks are established: (1) A Policy Neural Network, which receives the current timestep’s observation state and outputs the optimal policy for that timestep, driving the vehicle to perform the best action; (2) A Reward-based Value Network, used to evaluate the expected performance of the rewards for the policy adopted at the current timestep; (3) A Cost-based Value Network, used to assess the expected cost of the policy adopted at the current timestep. Figure 1 illustrates the robust highway autonomous driving decision framework based on RECPO, which includes the interaction data acquisition, preservation, and sampling (IDAPS) and the policy evaluation and optimization (PEO) components.

The task of the IDAPS section is to input the observed state at the current timestep into the three networks. This enables it to take the best action after receiving the optimal policy output from the policy network and to obtain the probability distribution of these actions under the current policy network. After taking an action, it receives feedback from the environment and itself, which includes the observed state, reward, costs at the current time step, and the state at the next time step. After successfully capturing the observation space at each step, it is saved in the Replay Buffer in the CMDP format. The training data, consisting of trajectories that sample data from the Replay buffer and incorporate data obtained from the current epoch, serve as the basis for optimizing the policy network and value networks.

For the PEO section, the task is to receive the observed states from IDAPS and pass on the optimal policy, the probability distribution of actions, and value estimates, thereby enabling it to take the best action and thus obtain training data (i.e., the trajectories). With this training data, PEO can update and optimize the policy network through RECPO, as well as update and optimize the value networks using mean squared error loss to maximize rewards within a safe range. The updated and optimized PEO then impacts IDAPS, with both components working together to facilitate iterative improvement.

## 4. Highway Vehicle Control Transformed into Safe Reinforcement Learning

This part will explore how to transform the highway autonomous driving decision-making task into a safe RL formal framework CMDP, including the definition of observation space, action space, reward function and cost function.

### 4.1. Constrained Markov Decision Process

The Markov Decision Process (MDP) is the standard form for modeling the operating environment of agents in the field of RL, providing a framework to describe environments in scenarios where outcomes are partly stochastic and partly under the control of the decision-maker. An MDP is defined as a tuple (S,A,R,P,μ), where *S* is the state space, representing all possible states of the environment; *A* is the action space, representing all possible actions that an agent can take; R:S×A×S→R, is a mapping from the Cartesian product of the state space and action space to real numbers, i.e., the reward function; P:S×A×S→[0,1], is the state transition probability function, where $P(s^{\prime }|s,a)$ is the probability of transitioning to state s’ from state s∈S by taking action a∈A; u:S→[0,1] is the initial state distribution; policy π:s→P(a) is defined as a mapping from states S to probability distributions over actions *A*; π(a|s) represents the probability of choosing action a in states. **ϕ** denotes the set of all feasible policies.

The goal of RL is to choose a policy π to maximize the expected value of the objective function J(π). The objective function J(π) is usually defined as the expected value of the cumulative reward under the policy π:

(1) $$J\left(\pi \right)≐\underset{\tau ∼\pi }{E}\left[\sum_{t=0}^{\infty }\gamma^{t}R\left(s_{t},a_{t},s_{t+1}\right)\right]$$

where, γ∈[0,1] is the discount factor; $\tau =(s_{0},a_{0},s_{1}\ldots )$ represents the trajectory; τ∼π represents that the distribution on the trajectory τ depends on $s_{0}∼\mu ,a_{t}∼\pi \left(\cdot |s_{t}\right),s_{t+1}∼P\left(\cdot |s_{t},a_{t}\right)$. Assuming R(τ) represents the discounted reward of trajectory τ, the policy value function can then be defined as: $V_{\pi }\left(s\right)≐E_{\tau ∼\pi }\left[R\left(\tau \right)|\right|s_{0}=s]$. And the policy behavior value function can be defined as: $Q_{\pi }\left(s,a\right)≐E_{\tau ∼\pi }\left[R\left(\tau \right)|\right|s_{0}=s,a_{0}=a)]$

Therefore, the advantage function $A_{\pi }$ can be defined as follows:

(2) $$A_{\pi }\left(s,a\right)=Q_{\pi }\left(s,a\right)-V_{\pi }\left(s\right)$$

CMDP is an extension of standard MDP that introduces a set of constraints, limiting the set of allowed strategies for this MDP. Specifically, CMDP defines a set of cost functions $C:C_{1},C_{2}\ldots C_{N_{c}}$, with limits constraints $d:d_{1},d_{2}\ldots d_{N_{c}}$. For every cost function $C_{i}$, a mapping S×A×S→R exists which maps transformation tuples to costs, similar to the reward function *R* in MDP. Currently, the cost value function is defined as: $V_{\pi }^{c}\left(s\right)≐E_{\tau ∼\pi }\left[C\left(\tau \right)|\right|s_{0}=s]$.And the policy behavior cost value function is defined as follows: $Q_{\pi }^{c}\left(s,a\right)≐\underset{\tau ∼\pi }{E}\left[C\left(\tau \right)|\right|s_{0}=s,a_{0}=a)]$

Therefore, the advantage function: $A_{\pi }^{c}\left(s,a\right)=Q_{\pi }\left(s,a\right)-V_{\pi }^{c}\left(s\right)$. The objective function J(π) of the policy π is updated to the expected discount report relative to the cost function: $J_{C_{j}}\left(\pi \right)=E\left[\sum_{t=0}^{\infty }\gamma^{t}C_{j}\left(s_{t},a_{t},s_{t+1}\right)\right]$. Therefore, the set of feasible security policies is updated to:

(3) $$\Pi_{C}=\left\{\begin{array}{c} \pi \in \varphi :\forall_{i},J_{C_{j}}\left(\pi \right)\leq d_{i} \end{array}\right\}$$

Therefore, compared to the ultimate goal of safety-awareness RL problems: π=argmaxJ(π). The final goal of the safe reinforcement learning problem is updated to:

(4) $$\pi^{*}=argmax_{\Pi_{c}}J\left(\pi \right)$$

It is considered that the policy optimization within the safety constraints is reasonable. Eitan Altman et al. showed that the set of all optimal policies for CMDP is included in the stationary policy [22].

### 4.2. Convert Decision-Making Problem for Autonomous Highway Driving into a Constrained Markov Decision Problem

This section first defines a CMDP to frame the problem of safe DRL. Next, the highway driving decision problem will be formalized as a CMDP by explicitly listing its key components, including the state space, action space, reward function, and safety cost function.

Section 3 provides a comprehensive problem definition, outlines the information observable by the EV, and describes the methodological framework. These elements facilitate the implementation of specific definitions.

Definition of the Observation Space: In a real-world highway environment, time is continuous, and the state space is infinite. Theoretically, to better describe the highway environment, it is advisable to capture as many dimensions of the observation space as possible to ensure our observations sufficiently describe the state. However, in practice, due to various factors including convergence speed, computational time, and complexity, to ensure the model’s real-time performance and efficiency, the observation space should focus attention on key information, similar to human perception. The observation space is defined as *S*:

$$S^{\prime }=\left(S_{EV}^{\prime },S_{SV}^{\prime },S_{EN}\right)$$

where, $S_{EV}^{\prime }$ is the observation of the agent’s own vehicle data; $S_{SV}^{\prime }$ is the observation of other vehicle data on the highway; $S_{EN}$ is other observation data around the environment.

**Figure 3:**
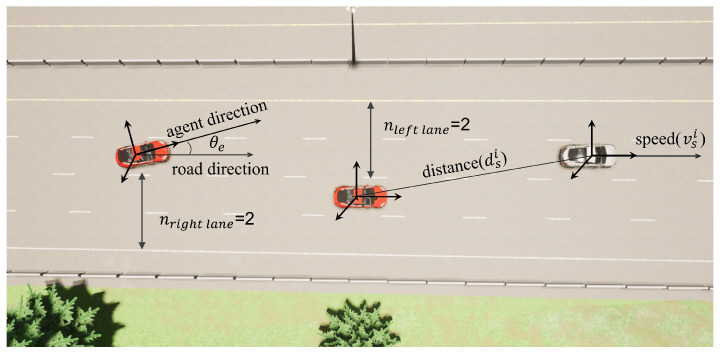
Definition of partial observation space.

For automobile control, the controller’s perception of the controlled object’s own data is indispensable. Therefore, we define $S_{EV}^{\prime }$:

$$S_{EV}^{\prime }=\left[t_{e},v_{e},\theta_{e},A_{t-1}\right]$$

where $t_{e}$ represents the current timestamp of the EV; $v_{e}$ represents the instant speed of the EV; $\theta_{e}=\psi_{e}^{v}-\psi_{e}^{r}$ represents the angle between the EV forward direction and the road direction; $A_{t-1}$ represents the time taken by the EV at the previous time step. The definition of $\theta_{e}$ is shown in Figure 3.

For $S_{SV}^{\prime }$, we define an Observation Zone that extends $d_{o}$ along the road in both the forward and backward directions from the EV, and laterally to the outer lanes on both sides. As shown in Figure 1, we divide the vehicles on the road into eight sections centered around the EV: front, rear, left, right, front-left, rear-left, front-right, and rear-right. Evidently, the number of vehicles in each section varies between busy and free road conditions. Due to these variations, the model cannot accept inputs from observation spaces of varying dimensions. Therefore, to ensure the stability of model training and the validity of observation space, we have chosen to use the observational data of the vehicles closest to the EV in each section to form the vehicle observation space. If no vehicles are observed in a section, it is filled with zero. Consequently, we define $S_{SV}^{\prime }$ as follows:

$$S_{SV}^{\prime }={\left\{S_{SV_{i}}^{\prime }\right\}}_{i=1}^{8}$$

where $SV_{i}$’s i∈[left,right,front,back,leftfront,leftback,rightfront,rightback]. For the simplicity and efficiency of the model, we define $S_{SV_{i}}$ for each part of the observation space as: $S_{SV_{i}}^{\prime }=\left[\begin{array}{c} e_{s}^{i},d_{s}^{i},v_{s}^{i} \end{array}\right]$; where $d_{s}^{i}$ represents the distance between the nearest vehicle $SV_{i}$ and EV in this part $d_{s}^{i}=\sqrt{{\left(x_{e}-x_{s}^{i}\right)}^{2}+{\left(y_{e}-y_{s}^{i}\right)}^{2}}$, $\left[x_{e},x_{s}^{i},y_{e},y_{s}^{i}\right]$ are the state spaces obtained from the environment, which are the position coordinates of the EV $\left(x_{e},y_{e}\right)$ and the position coordinates of the observation vehicle $\left(x_{s}^{i},y_{s}^{i}\right)$, respectively; $e_{s}^{i}$ represents whether this part of the orientation exists vehicle, $d_{s}^{i}<d_{o}$, then 1 otherwise 0; $v_{s}^{i}$ represents the speed of the nearest vehicle in this part in its forward direction. The definitions of $d_{s}^{i}$ and $v_{s}^{i}$ are shown in Figure 3.

In addition, the sub-observation space $S_{EN}$ that we define for the environmental state is consistent with the actual observed information in the problem definition:

$$S_{EN}=\left\{n_{l},n_{r}\right\}$$

It includes the number of left lanes $n_{l}$ and the number of right lanes $n_{r}$ that can describe the lane position of the EV and the total number of lanes. Their definitions are shown in Figure 3.

Definition of the Action space: For car control, the control usually only has longitudinal and lateral behaviors: (1) vertical behavior: accelerator or brake; (2) lateral behavior: lane change. In our study, the set behavior space has two dimensions: (1) lane change direction; (2) expected speed.

$$A=\left[a_{v},a_{c}\right]\;a_{i}\in \left[0,1\right]$$

For the steering action $a_{c}$, we quantify it into three behaviors: lane change to the left, lane change to the right, and lane keeping to simplify car control. The actual steering wheel angle and throttle/brake are generated by the PID controller we set up:

(5) $$u\left(t\right)=K_{p}e\left(t\right)+K_{i}\int_{0}^{t}e\left(t\right)dt+\frac{K_{d}de\left(t\right)}{dt}$$

where μ(t) is the control input at time *t*, such as accelerator, brake or steering wheel angle; e(t) is the error at time *t*, that is, the difference between the target value and the actual value. In this article, $e\left(t\right)=\{e_{v}\left(t\right),e_{c}\left(t\right)\}$ where $e_{v}\left(t\right)=v_{e}\left(t\right)-a_{v}\left(t\right),e_{c}\left(t\right)=\pm \sqrt{\Delta x^{2}+\Delta y^{2}}$, $v_{EV}^{t}$ and $a_{v}^{t}$ are the speed and target speed of EV at time *t*, respectively, Δx and Δy represent the differences in the x and y coordinates, respectively, between the electric EV’s position $\left(x_{e},y_{e}\right)$ and the target lane coordinates $\left(x_{l},y_{l}\right)$. The coordinates $\left(x_{l},y_{l}\right)$ are obtained from the waypoint in the chosen lane based on action $a_{c}$, which is the closest to a distance of approximately $d_{w}$ meters along the lane from the Electric Vehicle (EV). Specifically, in actual navigation, maps are utilized (or maps accessible within the simulator), each containing a series of node waypoints at the center of the lane for vehicle navigation. During the decision-making process, the EV identifies these waypoints and strategically selects a target waypoint in the target lane that is closest to $d_{w}$ meters along the lane, based on the action $a_{c}$ defined in this study. The selected waypoint’s absolute coordinates $\left(x_{l},y_{l}\right)$ on the map serve as the target lane coordinates for navigation. The $d_{w}$ is a hyperparameter, and its specific value in the experiment is shown in Table 1. $K_{p},K_{i},K_{d}$ are the proportional, integral and differential coefficients of PID controllers, these are hyperparameters that need to be adjusted according to the situation.

Definition of the Cost Function: In safe RL, the cost function is used to evaluate the costs associated with the actions of an agent, serving as the counterpart to the reward function. This function quantify the potential negative consequences of each action, thus aiding the agent in avoiding high-risk or inappropriate behaviors while pursuing rewards. The cost function in this study emphasizes enhancing safety in traffic scenarios and reducing potential collision risks. Specifically, the cost function includes the following components: (1) the cost of colliding with other objects, $c_{collision}$; (2) the cost of illegal lane changes, $c_{illegal}$; (3) the cost of driving off the road, $c_{out}$; (4) the cost of driving below the speed limit on highways, $c_{low\;speed}$; (5) the cost of maintaining too close a distance to surrounding vehicles, $c_{distance}$. Therefore, the cost function is defined as:

(6) $$C=c_{collision}+c_{out}+c_{illegal}+c_{low\;speed}+c_{distance}$$

**Figure 4:**
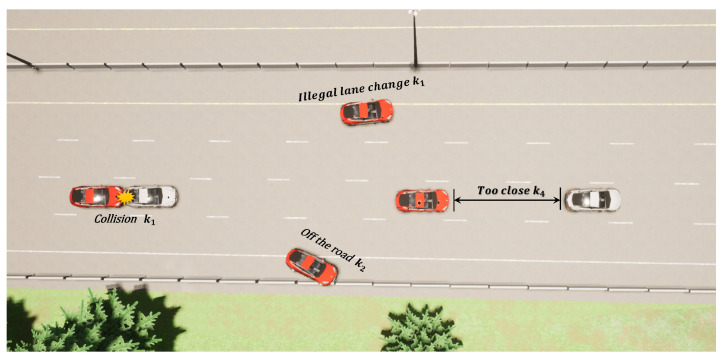
Definition of some sub-items of the cost function.

Once the EV collides with surrounding environmental objects (including vehicles, road edges, etc.) or changes lanes illegally, $k_{1}$ points of overhead will be added, as shown in Figure 4. On highways, a collision may lead to serious traffic accidents, so $k_{1}$ is usually a very large value:

$$c_{collision}=\left\{\begin{array}{c} k_{1},if\;Collision\\ 0,\;else \end{array}\right.$$

(7) $$c_{illegal}=\left\{\begin{array}{c} k_{1},if\;Illegal\\ 0,\;else \end{array}\right.$$

Likewise, being off the road can expose drivers and passengers to unpredictable dangers, and avoiding it is one of the top safety priorities. Therefore, $k_{2}$ will also be a maximum value, but the situation of leaving the road is more serious than a collision or illegal lane change, so it will be larger than $k_{1}$, giving the agent greater overhead to ensure that it even if a collision or illegal lane change occurs should also try not to trigger it as much as possible.

(8) $$c_{out}=\left\{\begin{array}{c} k_{2},if\;Out\\ 0,\;else \end{array}\right.$$

Obviously, the purpose of building highways is to provide vehicles with rapid passage to improve traffic efficiency. Therefore, there will be a minimum speed limit $v_{min}$ on the highway, and driving on the highway at a speed lower than the minimum speed limit increases the risk of traffic accidents. This principle is reflected in the cost function we studied: when the EV’s speed $v_{e}$ is lower than the road’s minimum speed limit $v_{min}$, it generates an overhead proportional to the difference between its own speed and the minimum speed $\left(v_{min}-v_{e}\right)$:

(9) $$c_{low\;speed}=\frac{k_{3}\left(v_{min}-v_{e}\right)}{v_{min}},if\;v_{e}<v_{min}$$

Maintaining a safe distance is a necessary condition for safe driving of the car. When the distance $d_{s}^{i}$ (that is, i=front) between the EV and the vehicle in front is less than the safe distance $d_{s}afe$, an overhead value of $k_{4}$ will be generated.

(10) $$c_{distance}=k_{4},if\;d_{s}^{i}<d_{safe}\;and\;e_{s}^{i}\;=\;1$$

Definition of the Reward Function: The reward function is the core concepts of reinforcement learning. This function maps states (or combinations of states and actions) to numerical rewards, thereby assessing the effectiveness of specific actions taken by an agent in a given state. It serves as the primary mechanism guiding the agent’s learning and actions, directly influencing the behavioral strategies formed during interaction with the environment. The ultimate goal of the agent is to maximize the total reward obtained in the environment while satisfying the constraints.

This study comprehensively considers driving efficiency and driver comfort designing the reward function. Therefore, the reward function of this study includes: (1) Driving efficiency reward $r_{efficiency}$; (2) Driving comfort reward $r_{comfort}$; (3) Driving safely to the end of the road reward $r_{finish}$; (4) Penalty *C* obtained from the cost function. Define the reward function as follows:

(11) $$R=r_{efficiency}+r_{comfort}+r_{finish}-C$$

In this study, efficiency refers to traveling safely and as quickly as possible on the highway within the legal limits to reach the destination. Therefore, we set the efficiency part of the reward function: If the EV’s speed $v_{e}$ is within the road regulations, it can obtain a reward inversely proportional to the difference between its own speed and the maximum speed $v_{max}$:

(12) $$r_{efficiency}=\left\{\begin{array}{c} \frac{k_{5}\left(v_{e}-v_{min}\right)}{v_{max}-v_{min}},if\;v_{e}\in \left[v_{min},v_{max}\right]\\ \;-k_{5} \end{array}\right.$$

This study considers a universal autonomous driving framework, so systems such as suspension, seats, and temperature control that affect comfort are not taken into account. At this time, the comfort of the vehicle is mainly reflected in the magnitude of instantaneous acceleration. We encourage EVs to drive with smaller acceleration. When the maximum instantaneous acceleration $a_{e}$ of EV is greater than the set value $a_{max}$ (generally 3 m/s^2^), a penalty will be given. Otherwise, a reward will be given:

(13) $$r_{comfort}=\left\{\begin{array}{c} -k_{6}min\left(\frac{a_{e}}{10},1\right),if\;a_{e}>a_{max}\\ \;k_{6}min\left(1-\frac{a_{e}}{a_{max}},1\right),else \end{array}\right.$$

where $a_{e}$ is the acceleration of EV, defined as: $a_{e}=\frac{v_{e}\left(t\right)-v_{e}\left(t-1\right)}{t_{step}}$, $t_{step}$ is time step size.

The fundamental purpose of this study is to safely pass the road. Therefore, for successfully completing the set road safely, we will give the EV reward $k_{7}$, and $k_{7}$ is generally a maximum value:

(14) $$r_{finish}=k_{7},if\;Finish=True$$

$k_{1},k_{2},k_{3},k_{4},k_{5},k_{6},k_{7}$ are all settable weight hyperparameters. After establishing the reward function, cost function, observation space, and action space, we can define our highway autonomous driving CMDP. During the IDAPS phase, the environment is explored based on the established model to obtain trajectories $\tau =\{s_{t},a_{t},s_{t+1},r_{t},c_{t}\ldots \}$, which are then stored in the Replay buffer for use by the PEO section.

## 5. Autonomous Highway Driving Based on Safe Reinforcement Learning

According to the CMDP model of highway autonomous driving decision-making problem constructed in Section 4, the EV interacts with the environment through the IDAPS component, samples and storing trajectories into the Replay buffer. The PEO component retrieves training data from the Replay buffer and IDAPS, using this data to update and optimize strategies based on RECPO. Specifically, The PEO obtains the probability distribution of each segment of training data at the time of sampling and calculates importance sampling weights based on the current policy’s probability distribution. These weights are used to adjust parameters allowing training data from old policies can be applied to the evaluation and optimization of the current new policy. The optimized policy is applied to IDAPS for testing and sampling data. This process is repeated in cycles, iterating until the policy meets performance requirements.

In this section, we first delve deeply into the use of importance sampling technology within the RECPO algorithm. Importance sampling is crucial in online learning scenarios because it addresses the issue of previously collected experience data becoming obsolete due to updates in policy. This technique optimizes data reuse and enhances learning efficiency by assigning different sampling weights to various data points, thereby accelerating the model’s convergence speed.

Next, we explore the policy optimization process of RECPO. As an innovative safe reinforcement learning algorithm, RECPO is designed not only to maximize rewards but also to ensure that the strategy’s safety and performance meet specific standards. Additionally, RECPO possesses a unique capability to utilize historical policy experience for updating parameters. This mechanism not only enhances the utilization rate of old data but also significantly improves the adaptability and robustness of the algorithm.

By implementing these technical features, RECPO effectively mitigates issues of catastrophic forgetting (where the model forgets old knowledge when learning new tasks) and the long-tail problem (where parts of the data are difficult for the model to learn due to their very low frequency of occurrence). In summary, through these advanced integrations, RECPO not only optimizes the learning process but also ensures the strategy’s comprehensiveness and safety, providing an efficient and reliable solution for reinforcement learning tasks in complex environments.

### 5.1. Importance Sampling

The core of importance sampling is to capture the discrepancy between experiences under an old policy and a new policy, that is, to compute a weight $w_{t-t^{\prime }}\left(\tau_{t^{\prime }},\pi_{t}\right)$ that describes the impact of the trajectory $\tau_{t^{\prime }}$ from the old policy on updating the parameters of the new policy model $\pi_{t}$. In IDAPS, the EV first obtains the observation space $S_{t}$ at time *t*. At this stage, the PEO is in the inference phase, mapping the $s_{t}$ obtained by IDAPS to the action space to determine the action $a_{t}$ at time *t*. After the EV executes action $a_{t}$, the environment is influenced to transition from $s_{t}$ to $s_{t+1}$, and the agent receives feedback from the environment $\left[r_{t},c_{t},f_{t}\right]$ (where $f_{t}$ at time *t* indicates whether the agent has safely completed that segment of the road, false if yes and true otherwise). For traditional RL, the trajectory obtained at this time would be $\tau_{t}^{\prime }=\left[s_{t},a_{t},s_{t+1},r_{t},c_{t},f_{t}\right]$, and in non-online learning algorithms, a batch $B_{buff}^{\prime }=\left[\tau_{0}^{\prime },\tau_{1}^{\prime }\ldots \tau_{N_{\tau }}^{\prime }\right]$ is obtained at the end of each epoch.

(15) $$P\left(a_{t},s_{t}\right)=\frac{1}{\sqrt{2\pi \sigma_{t}^{2}}}e^{-\frac{{\left(a_{t}-\mu \left(s_{t}\right)\right)}^{2}}{2\sigma_{t}^{2}}}$$

where $\mu (s_{t})$ and $\sigma_{t}^{2}$ represent the mean and variance of the policy network in state $s_{t}$ at time *t*, and they define the distribution of behavior in a given state.

For discrete space, the strategy is usually modeled as a softmax distribution, in which case $P(a_{t},s_{t})$ is defined as:

$$P\left(a_{t},s_{t}\right)=\frac{e^{f(a_{t},s_{t})}}{\sum_{a^{\prime }}e^{f(a^{\prime },s_{t})}}$$

where f(a,s) represents the score output by the policy network for each behavior. Usually the softmax function will ensure that the sum of the probabilities of all possible actions is 1: $\sum_{i=0}^{n}p\left(a_{t}^{i},s_{t}\right)=1$

In importance sampling, we typically need two distributions: (1) Target distribution $Q(a_{t},s_{t})$: used for sampling to estimate the expected value of a certain quantity; (2) Proposal distribution $P(a_{t},s_{t})$: the distribution from which we actually sample, which should be similar to the target distribution. The core idea of importance sampling is to use samples from the proposal distribution *P* to estimate the expectations under the target distribution *Q*. Because sampling directly from *Q* may be difficult or computationally expensive, all sampled data are reweighted to reflect their probability distribution in *Q*. These weights are then used to evaluate the transition from *P* to *Q*. When estimating the expected value of a function f(x) under the target distribution *Q*, $E_{Q}\left[f\left(x\right)\right]$, we can draw samples $x_{j}$ from *P* and apply importance weights $w(x_{j})$ to them.

(16) $$w\left(x_{j}\right)=\frac{Q(x_{j})}{P(x_{j})}$$

These weights adjust the contribution of each sample, and we can get the expectation of samples drawn from the *P* distribution under the *Q* distribution:

$${\hat{E}}_{Q}\left[f\left(x_{j}\right)\right]\approx \frac{1}{N_{s}}\sum_{j=1}^{N_{s}}w\left(x_{j}\right)f\left(x_{j}\right)$$

where $N_{s}$ represents the sample number.

Therefore, for importance sampling in RECPO, we can obtain the importance weight $w_{t-t^{\prime }}$ of the trajectory $\tau_{t^{\prime }}$ of the old policy $\pi_{t^{\prime }}$ at time t-n under the new policy $\pi_{t}$:

(17) $$w_{t-t^{\prime }}\left(\tau_{t^{\prime }},\pi_{t}\right)=\frac{P_{t}\left(a_{t^{\prime }},s_{t^{\prime }}\right)}{P_{t^{\prime }}\left(a_{t^{\prime }},s_{t^{\prime }}\right)}$$

Thus, at each timestep t, we need to obtain a trajectory $\tau_{t}=\left[s_{t},a_{t},s_{t+1},r_{t},c_{t},f_{t},p_{t}\right]$. The Replay buffer ultimately comprises $B_{buff}=\left[\tau_{0},\tau_{1}\ldots \tau_{N_{\tau }}\right]$. During the training process following inference, samples $\tau^{b}$ will be drawn from $B_{buff}$. The sum of the extracted samples $\tau^{b}$ and the samples $\tau^{n}$ sampled during inference before the current policy update is the actual sample $\tau =\tau^{n}\cup \tau^{b}$ sampled for policy optimization. This ensures that the policy model reflects the actual situation in real-time and also captures past situations, especially scenarios not present in $\tau^{n}$ but existing in $\tau^{b}$, effectively mitigating the catastrophic forgetting problem in RL. Simultaneously, the continual random resampling of old data facilitates the efficient reuse of long-tail data, which might be rare in actual inference, thus alleviating the long-tail problem.

### 5.2. Evaluation and Optimization of Policy Networks

In the previous section, we introduced importance sampling. In this section, we first discuss the application of importance sampling techniques in policy updates, and then we describe policy optimization based on trust regions and safety zones, along with their respective meanings. Subsequently, we will explore the optimization methods adopted for different scenarios. Finally, we will elaborate on the techniques used to update policy model parameters. Generalized Advantage Estimation (GAE) is a technique used to estimate the advantage function, which helps us identify the potential additional value of choosing a specific action compared to following the average action selected under the current policy. GAE is commonly used in policy gradient algorithms to enhance the efficiency and stability of policy updates. Let $A^{\pi }\left(s,a\right)$ represent the advantage of taking action *a* in state *s* relative to the average performance of policy π. GAE estimates the advantage function by combining a weighted average of multiple temporal difference (TD) errors. $A^{\pi }\left(s_{t},a_{t}\right)$ is defined as follows:

(18) $$A^{\pi }\left(s_{t},a_{t}\right)=\sum_{l=0}^{T-t-1}{\left(\gamma \lambda \right)}^{l}\delta_{t+l}^{V}$$

where $\delta_{t}^{V}$ is the TD residual at time *t*, defined as: $\delta_{t}^{V}=r_{t}+\gamma V^{\pi }\left(s_{t+1}\right)-V^{\pi }\left(s_{t}\right)$; $r_{t}$ represents the immediate reward received after taking action $a_{t}$ in state $s_{t}$; $V^{\pi }\left(s\right)$ is the value function of state *s* under policy π; γ is the discount factor used to balance the importance of immediate rewards and future rewards; λ is the Lagrange multiplier; *T* represents the maximum time step number of the trajectory, and *l* is used to index data starting from time *t*.

If the experience from an old policy is introduced during training, the advantage function will be affected by the distance between the policy model distributions. Specifically, when incorporating experiences from an old policy into a new one, it is necessary first to obtain importance weights $w_{i}$ to assess this impact and ensure that the old policy is correctly utilized in the new policy. To prevent numerical underflow (i.e., computational issues caused by multiplying very small numbers), the calculation of $w_{i}$ in RECPO’s policy optimization uses the log probability distribution:

(19) $$w_{t-t^{\prime }}\left(\tau_{t^{\prime }},\pi_{t}\right)=\frac{logP_{t}\left(a_{t^{\prime }},s_{t^{\prime }}\right)}{logP_{t^{\prime }}\left(a_{t^{\prime }},s_{t^{\prime }}\right)}$$

For old experience used in new strategies, its advantage function takes into account the impact of strategy changes, and its advantage function definition is updated to $A^{\pi }\left(s_{t},a_{t}\right)$:

(20) $$A^{\pi }\left(s_{t},a_{t}\right)=\sum_{l=0}^{T-t-1}{\left(\gamma \lambda \right)}^{l}\delta_{t+l}^{V}w_{t-t^{\prime }}\left(\tau_{t^{\prime }},\pi_{T}\right)$$

We use formal parameters $\theta_{\pi }$ to characterize the current policy $\pi_{k}$. The policy parameters of the updated policy $\pi_{k+1}$ are $\theta_{k+1}$. If the update amount of the policy is Δθ, the policy update is defined as:

(21) $$\theta_{k+1}=\theta_{k}+\Delta \theta$$

The following discusses focuses on how to determine the policy update amount Δθ. Not every policy update violates safety constraints; we use the trust region method to update policies that do not violate safety constraints. For policy updates that do violate safety constraints, it is necessary to solve the dual problem to find a policy that maximizes the expected reward without violating the safety constraints. For cases where the policy update amount exceeds the trust region and still does not meet safety constraints, the policy is updated using the natural gradient.

From Equation (1), the objective function $J_{c}\left(\pi \right)$ after considering the cost function:

(22) $$J_{c}\left(\pi \right)≐\sum_{\tau ∼\pi }\left\lfloor \sum_{t=0}^{\infty }\gamma^{t}C_{i}\left(s_{t},a_{t},s_{t+1}\right)\right\rfloor$$

where $C(s_{t},a_{t},s_{t+1})$ is the cost function, which represents the cost of taking action $a_{t}$ in the $s_{t}$ state to change the state to $s_{t+1}$. On the basis of obtaining J(π) (refer to Equation (1)) and $J_{c}\left(\pi \right)$ (refer to Equation (22)), we can calculate the neural network gradient $\hat{g}$ based on the reward value function and the neural network gradient $\hat{b}$ based on the cost value function:

(23) $$\hat{g}=\nabla_{\theta }J\left(\pi \right)=\frac{1}{N_{\tau }}\sum_{j=0}^{N_{\tau }}\sum_{t=1}^{N_{t}}\nabla_{\theta }E_{\tau_{\pi_{k}}∼\pi_{\theta }}\left[log\pi_{\theta }\left(a_{t}|s_{t}\right)A^{\pi }\left(s_{t},a_{t}\right)\right]$$

(24) $$\hat{b}=\nabla_{\theta }J_{c}\left(\pi \right)=\frac{1}{N_{\tau }}\sum_{j=0}^{N_{\tau }}\sum_{t=1}^{N_{t}}\nabla_{\theta }E_{\tau_{\pi_{k}}∼\pi_{\theta }}\left[log\pi_{\theta }\left(a_{t}|s_{t}\right)A_{c}^{\pi }\left(s_{t},a_{t}\right)\right]$$

where $N_{\tau }$ represents the number of trajectories τ; $N_{t}$ represents the total time step. As mentioned earlier, different policies will lead to different safety levels. Some policies are relatively safe and some policies are relatively dangerous, especially if they seriously violate the “safety domain” we have established. Therefore, we define the policy safety level judgment factors $\hat{c}$ and *B*, which are used to evaluate the safety level of the policy to use the appropriate policy update method:

(25) $$\hat{c}=E\left[\sum_{t=0}^{T}\gamma^{t}C_{i}\left(s_{t},a_{t},s_{t+1}\right)\right]-\frac{c_{d}}{1-\gamma }$$

(26) $$B=\delta -\frac{{\hat{c}}^{2}}{{\hat{b}}^{T}H^{-1}\hat{b}}$$

where *H* is the constraint-based Hessian matrix; $c_{d}$ is the overhead constraint threshold, which is a hyperparameter; δ is the policy update step adjustment factor, which is a hyperparameter. In order to ensure that the policy update pace remains within a safe range during the policy iteration process, avoid performance mutations caused by excessive updates, and ensure the stability of the learning process, the trust region method based on Kullback Leibler (KL) divergence is used in the policy update. The definition as a constraint is:

(27) $$E_{\left(a,s\right)∼\pi_{old}}[D_{KL}(\pi_{old}\left(a|s\right)||\pi_{\theta }\left(a|s\right))]\leq \delta_{KL}$$

where $\theta_{KL}$ is the set KL dispersion threshold; $D_{KL}$ is the dispersion size, which is defined as: $D_{KL}(\pi_{old}\left|\right|\pi_{\theta })=\int \frac{\pi_{old}\left(a|s\right)log\pi_{old}\left(a|s\right)}{\pi_{\theta }\left(a|s\right)}da$

When the policies are both within the safety domain and the trust domain, that is, $∥\hat{b}∥<1\times 10^{-8}\;\&\;\hat{c}<0\;or\;\hat{c}<0\;\&\;B<0$, there is no need to guide the policy through safety constraints, so it is based on constraint restrictions. The problem is similar to the traditional policy update problem. We can obtain policy update:

(28) $$\theta_{k+1}=\theta_{k}+\sqrt{\frac{{\hat{g}}^{T}H^{-1}\hat{g}}{\delta }}H^{-1}\hat{g}$$

where $\hat{g}$ represents the gradient of the value function.

When the trust domain of the policy is not all included in the safety domain, that is, B>0, the policy at this time may make some illegal and safety behaviors, so it is necessary to guide policy updates through safety constraints. At this time, the safety domain and the trust domain have an intersection, and the convex optimization problem can be solved. The conjugate gradient method is used to solve the inverse application problem of the Hessian matrix of KL divergence, and the policy update is obtained:

(29) $$\theta_{k+1}=\theta_{k}+\frac{1}{\lambda^{*}}H^{-1}\hat{g}-\frac{v^{*}}{\lambda^{*}}H^{-1}\hat{b}$$

Among them, $\hat{b}$ represents the constraint gradient, which is obtained by Equation (24); $\lambda^{*}$ is an adjustable Lagrange multiplier, the purpose is to find a λ⩾0 to maximize the objective function value; $v^{*}$ is the “safety margin” metric adjustment”, which expresses how the calculated adjusted $\lambda^{*}$ affects the balance of rewards and constraint violations. If $\lambda \hat{c}$ (adjusted constraint violation) is greater than r, it means that $v^{*}$ must be adjusted to remain safe. If the calculation is negative (i.e., the reward exceeds the adjusted constraint violation), $v^{*}$ will be zero, indicating no further adjustment is needed.

$$\lambda^{*}=argmax_{\lambda \geq 0}\left\{\begin{array}{cc} f_{a}\left(\lambda \right)≜\frac{1}{2\lambda }\left(\frac{r^{2}}{S_{l}}-q\right)+\frac{\lambda }{2}\left(\frac{{\hat{c}}^{2}}{S_{l}}-\delta_{KL}\right)-\frac{r\hat{c}}{S_{l}} & if\;\lambda \hat{c}-r>0\\ f_{b}\left(\lambda \right)≜-\frac{1}{2}\left(\frac{q}{\lambda }+\lambda \delta_{KL}\right) & else \end{array}\right.$$

(30) $$v^{*}={\left(\frac{\lambda^{*}\hat{c}-r}{S_{l}}\right)}_{+}$$

where $S_{l}={\hat{b}}^{T}H^{-1}\hat{b}$. After obtaining $\lambda^{*}$ and $v^{*}$, use the conjugate gradient method to calculate the direction $x_{k}$ when the policy is updated: $x_{k}=H^{-1}\left(g-\hat{b}\nu^{*}\right)$.

At this point we can get the current policy update parameters:

(31) $$\theta_{k+1}=\theta_{k}+\frac{\alpha }{\lambda^{*}}x_{k}\approx \theta_{k}+\frac{1}{\lambda^{*}}H^{-1}\hat{g}-\frac{v^{*}}{\lambda^{*}}H^{-1}\hat{b}$$

α is obtained by Backtracking Line Search.

When the policy goes in a direction that cannot lead to the safe domain, at this time $\hat{c}>0\;\&\;B<0$, no matter how we guide it, it cannot guarantee its security, and the security constraints cannot take effect at this time. Therefore, we must roll back the strategy or modify the strategy to return it to a direction that can lead to the safe region. At this time, the convex optimization problem is unsolvable. We use natural gradients to update the strategy:

(32) $$\theta_{k+1}=\theta_{k}+\alpha_{lr}\tilde{\nabla }J_{F}\left(\theta \right)=\theta_{k}-\sqrt{\frac{2\delta }{{\hat{b}}^{T}H^{-1}\hat{b}}}H^{-1}\hat{b}$$

where $\alpha_{lr}$ is the learning rate; $\tilde{\nabla }J_{F}\left(\theta \right)$ is the natural gradient, obtained by preconditioning the regular gradient $\Delta J_{F}\left(\theta \right)$ to the inverse of the F Fisher Information Matrix, which is defined as: $\tilde{\nabla }J_{F}\left(\theta \right)=F^{-1}\nabla J_{F}\left(\theta \right)$ and *F* is defines as: $F=E_{\tau ∼\pi_{\theta }}\left[\nabla_{\theta }\log\pi_{\theta }\left(a|s\right)\nabla_{\theta }\log\pi_{\theta }{\left(a|s\right)}^{T}\right]$.

Through this part, we divide the safety of the policy in the update optimization process into three situations to choose the optimization update method of the policy, so that the policy update can ensure the safety of the policy while maximizing the reward.

### 5.3. Value Network Parameter Update

Network updating is a crucial part of the optimization process. The value network is usually used to predict the expected return under a given state, which helps guide the policy network to learn and make decisions more effectively. The accuracy of the value network significantly impacts the decision-making of the policy network. Below we introduce the update process for the value network. As mentioned earlier, when the value network is updated, the trajectory τ under the policy network output policy π is obtained from IDAPS, including state $s_{t}$, action $a_{t}$, reward $r_{t}$, cost $c_{t}$, next state $s_{t+1}$, etc. For each state $s_{t}$, the agent can have a reward $R_{t}$ and a cost $C_{t}$.

Updating the parameter ϕ and ψ of the value network is achieved by minimizing the difference between the predicted value and the actual calculated return, and we implement it using the Root Mean Square Error (MSE) loss function, defined as:

$$L\left(\phi \right)=E_{s_{t}∼\pi }\left[{\left(V_{\phi }\left(s_{t},a_{t}\right)-R_{t}\right)}^{2}\right]$$

(33) $$L\left(\psi \right)=E_{s_{t}∼\pi }\left[{\left(C_{\psi }\left(s_{t},a_{t}\right)-C_{t}\right)}^{2}\right]$$

Finally, we use a variant of gradient descent (the Adam optimizer) to adjust the network parameters to minimize the loss functions L(ϕ) and L(ψ):

$$\phi \leftarrow \phi -\alpha_{lr}\nabla_{\phi }L\left(\phi \right)$$

(34) $$\psi \leftarrow \psi -\alpha_{lr}\nabla_{\psi }L\left(\psi \right)$$

### 5.4. Highway Automatic Driving Algorithm Based on RECPO

This section introduces our proposed RECPO algorithm, which ensures the improvement of reward performance and the satisfaction of security cost constraints, as shown in Algorithm 1. The RECPO algorithm initially initializes the parameters of the policy, reward, and safety value neural networks, along with other necessary experimental parameters. Subsequently, it interacts with the environment using the initialized policy. For the autonomous vehicle, it receives state space information from the environment; the policy neural network guides the action space, directing the vehicle into the next state. After completing a round of interaction with the environment, the vehicle stores the trajectory information collected during this interaction in the Replay buffer. The policy neural network then extracts a specified batch size of trajectory information from the Replay buffer for optimization. When the control policy is at a high safety level, the optimization is performed using the Equation (28); at a medium safety level, the Equation (29) is used; at a lower safety level, a linear backtracking method (32) is employed to minimize the safety value. Finally, a gradient-based method is used to update the parameters of the reward and safety value neural networks to minimize the gap between estimated and actual values.
**Algorithm 1** Replay Buffer Constrained Policy Optimization**Input:** Initialize $\pi ,V_{r},V_{c},\phi ,\psi$, set $cost_{d},\gamma ,kl_{m}ax,N_{e}$ **for** epoch $n=0,1,2,3\ldots \to N_{e}$ **do**       **for** t=0,1,2…→ done is True **do**          Collect $s_{t}$ when interacting with the environment in IDAPS          Collect $a_{t}=s_{t}\to V_{r}\left(s_{t}\right)\;\&\;V_{c}\left(s_{t}\right)\to \pi \left(s_{t}\right)$          $s_{t+1}⇐s_{t}$, get the feedback reward $r_{t}$, cost $c_{t}$, done and the probability distribution $P_{t}\left(a_{t},s_{t}\right)$ of the policy network $\pi_{t}$ for $a_{t}$          Obtain trajectory $\tau_{t}=\left[s_{0},a_{0},r_{0},c_{0},P_{0}\left(a_{0},s_{0}\right),s_{1}\ldots \right]\to$ Replay Buff     **end for**     **for** do k=0,1,2…          Sampling the trajectory *D* from the Replay Buffer          Calculate importance weight *w*          Calculate advantage function of reward and cost function: ${\hat{A}}^{\pi }\left(s,a\right),{\hat{A}}_{c}^{\pi }\left(s,a\right)$          Calculate $\hat{g},\hat{b},\hat{c},\hat{B}$:                    $\hat{g}=\nabla J\left(\pi \right),\hat{b}=\nabla J_{c}\left(\pi \right)$                    $\hat{c}=\sum_{t=0}^{T}\gamma^{t}C_{t}+\frac{d_{i}}{1-\gamma }$                    $B=\delta -\frac{{\hat{c}}^{2}}{{\hat{b}}^{T}H^{-1}\hat{b}}$          **if** $\left(∥\hat{b}∥<1\times 10^{-8}\;\&\;\hat{c}<0\right)$ or $\left(\hat{c}<0\;\&\;B<0\right)$ **then**                update policy network as:                    $\theta_{k+1}=\theta_{k}+\sqrt{\frac{{\hat{g}}^{T}H^{-1}\hat{g}}{\delta }}H^{-1}\hat{g}$ //see the Equation (28)           **else if** B>0 **then**             solve convex dual problem, get $\nu^{*},\lambda^{*}$             solve α by backtracking line search, update policy network as:                    $\theta_{k+1}=\theta_{k}+\frac{\alpha }{\lambda^{*}}H^{-1}\left(\hat{g}-\hat{b}\nu^{*}\right)$ // see the Equation (29)           **else if** $\hat{c}>0\;\&\;B<0$ **then**              update policy network as:                    $\theta_{k+1}=\theta_{k}-\sqrt{\frac{2\delta }{{\hat{b}}^{T}H^{-1}\hat{b}}}H^{-1}\hat{b}$ // see the Equation (32)           **end if**           Update ϕ,ψ as:                    $\phi =argmin_{\phi }E\left[{\left(V_{r}\left(s_{t}\right)-{\hat{R}}_{t}\right)}^{2}\right]$                    $\psi =argmin_{\psi }E\left[{\left(V_{c}\left(s_{t}\right)-{\hat{C}}_{t}\right)}^{2}\right]$     **end for** **end for**

## 6. Experiment

### 6.1. Experimental Setup

In this study, to test the effectiveness, robustness, and safety of the proposed algorithm (RECPO), we used the Carla simulation platform to construct a highway driving scenario. I conducted experiments on several models in the same scenario, including the current advanced DDPG-based highway autonomous driving strategy [12], IDM [51] + MOBIL [52], and the traditional CPO algorithm [25]. We focused on vehicle safety, comfort, and driving efficiency during the testing process.

On the Carla simulation platform version 0.9.14, this study constructed a training scenario consisting of a three-lane highway with lanes 3.5 m wide. The RECPO and traditional CPO models in this project were developed using the PyTorch framework, while the DDPG model was built on the TensorFlow framework. This study used an NVIDIA GeForce RTX 3070Ti GPU and the Ubuntu 20.04 operating system. Using built-in sensors and the Python API of the Carla simulator, this study captured vehicle interaction data and controlled vehicle behavior, converting vehicle actions (such as speed, lane changes, collision signals) into data signals, and translating control commands into car motion instructions, such as converting acceleration commands into throttle, brake, and steering angle in the simulation.

Due to specific needs for the highway, existing scenarios in Carla could not meet the experimental requirements, so a 10-km long, 3.5-m wide three-lane rectangular road was customized using RoadRunner R2022b software. In each simulation, a 1-km long straight road segment was selected for training, where every 50 m, 3 vehicles were arranged for simulation. To ensure the real-time nature of the simulation, the Carla simulation was set to synchronous mode, with control cycles and scenario timestep both set at 0.05 s. Each vehicle’s target speed was set to 25 m per second, with speed disturbances in the range of [−5, 5] added during driving to simulate real driving speed variations. Moreover, in each simulation round, some vehicles would randomly change lanes within a safe range to simulate human driving behavior.

The policy and value neural network structures of our RECPO algorithm and the traditional CPO algorithm were set as 31×128×128×2 and 31×128×128×1 MLPs, respectively, using the tanh activation function and the Adam optimizer. For the DDPG algorithm, we replicated the original settings as closely as possible; its actor and critic neural network structures were set as 31×400×300×2 and 31×400×300×1 MLPs, respectively, using the relu activation function and the Adam optimizer. Each simulation ran for a maximum of 100 time steps, collecting up to 32×100 samples. The learning rate started from 1 × $10^{-3}$ and linearly decayed to 0. The training algorithms stopped after a maximum of 5000 iteration updates. The main parameters of the experiment are shown in Table 1.

### 6.2. Experimental Training Process

**Figure 5:**
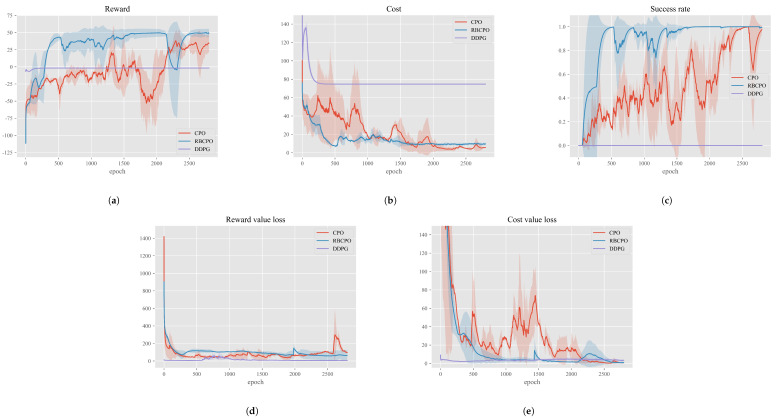
Training curve—The shaded area of each color line in all graphs represents its standard deviation. Actor loss is plotted in Reward value loss, critic loss is plotted in Cost value loss. The cost of DDPG is quantified according to the standards of RECPO and CPO.

In this experiment, we trained three Highway autonomous driving decision-making policies using RECPO, CPO, and DDPG algorithms and compared their rewards, overheads, and decision-making performance in terms of safety, efficiency, and comfort. To form a sufficient contrast, CPO utilized the same reward function, cost function, and hyperparameters as ours. For DDPG, which operates on an actor–critic structure, we adopted the parameters from [12] to ensure it had a sufficient performance baseline for comparison. Figure 5 shows the performance comparison of the three algorithms during training.

The training reward trends of the three algorithms are shown in Figure 5a. Both CPO and RECPO exhibit a clear upward trend in rewards during training, while DDPG struggles to show an increase. This could be partly because the reward punishment basis in DDPG’s reward function is smaller compared to CPO and RECPO, making its trend appear flatter. Additionally, the issue may be due to the direct application of hyperparameter settings from [12] to our more complex scenario without fine-tuning. Clearly, DDPG performs poorly in terms of reward in this scenario. Our focus is more on comparing RECPO with CPO, as they share more consistent settings on multiple hyperparameters, better illustrating the performance improvement of our RECPO algorithm relative to CPO. From the graph, it is evident that RECPO quickly improves around the 300–500 epoch range, reaching a very high reward value near 1500 epochs and approaching convergence. In contrast, CPO’s reward convergence rate appears slow. Before 2000 epochs, CPO’s reward rises very gradually, and it is only around 2300 epochs, when RECPO’s reward has already converged, that CPO begins to rapidly increase, equivalent to around 300 epochs of RECPO. The rising phase of the reward indicates that the ego has found the correct direction for strategy optimization; during this time, the cost drops quickly, and the strategy meets safety conditions allowing the ego to start maximizing rewards. Clearly, Figure 5a demonstrates the performance advantage of RECPO over CPO and DDPG. With the help of the importance sampling experience replay pool, it can find the appropriate strategy optimization direction and approach convergence more quickly.

Figure 5b shows the changes in costs during the training process for RECPO, CPO, and DDPG. In the CPO and RECPO algorithms, a cost surrogate rather than actual cost is used for safety assessment in policy evaluation, but the cost is the data that truly represents the ego’s actual performance in the environment, and the cost surrogate is closely related to it. Therefore, this section will only discuss the cost in the training processes of the CPO and RECPO algorithms. The trend in the graph shows that around the 500th epoch, the RECPO algorithm found a better policy optimization direction, causing the cost surrogate to decrease rapidly, which also quickly reduced the actual performance cost in the scenario. Eventually, both the CPO and RECPO algorithms were able to reduce the cost to a lower level by 2500 epochs (when the cost is below 15, there is no possibility of hazardous behavior in our scenario, only the possibility of potentially hazardous situations, such as being closer than a safe distance to the vehicle in front). This indicates that both the CPO and RECPO algorithms perform excellently in terms of safety in the scenario.

Success rate is an important indicator that describes the completion of safe driving by the ego vehicle during a simulation training round. Figure 5c shows the changes in success rates during the training processes of RECPO, CPO, and DDPG algorithms. As can be seen from the figure, the success rate of DDPG has almost no upward trend and the reward is very low with the overhead. This indicates that DDPG explores the scene to a very low extent, and since DDPG lacks specific parameters that limit safety, very low rewards imply extremely unsafe behavior on the part of the self. This is also highlighted by its high cost quantified by the overhead functions of RECPO and CPO. The success rate curves for RECPO and CPO demonstrate their superior learning capabilities. Comparatively, RECPO’s success rate rises much faster than CPO and eventually converges to 1. This validates the effectiveness of the importance sampling-based Replay buffer proposed in our study, which enables the network to learn quickly and converge effectively.

The loss function describes the gap between the network’s estimated values and the actual values, with smaller loss indicating more accurate network evaluations. Figure 5d and Figure 5e shows the loss values for the value functions of RECPO and CPO, as well as the critic network for DDPG during the training process. From the trend in the graph, the evaluation capabilities of the networks for all three algorithms improve during simulation training. Notably, both the RECPO and CPO algorithms exhibit a sharp spike in loss after a higher number of training rounds. This could be due to catastrophic forgetting or because the ego encountered previously unseen scenarios. However, the graph shows that the spike in RECPO is much smaller than in CPO, demonstrating the effectiveness of our RECPO in combating catastrophic forgetting and addressing long-tail data issues.

### 6.3. Performance Comparison of RECPO, CPO, DDPG and IDM + MOBIL after Deployment

To evaluate the performance of different algorithms, we conducted tests in the same scenario. To better assess each algorithm’s performance, we used eight indicators to quantify the efficiency, comfort, and safety aspects of each EV: (1) Efficiency, which includes average speed and the standard deviation of speed; (2) Comfort, measured by average acceleration, standard deviation of acceleration, and average jerk; (3) Safety, which includes success rate, the number of times safety distance was triggered, and following distance. Specific data are shown in Table 2, Table 3 and Table 4.

In terms of driving efficiency, RECPO and CPO demonstrated high and similar levels during testing. The maximum road speed limit is 30 m/s, with CPO and RECPO achieving speeds of 27.3 m/s and 27.5 m/s, respectively, and both maintaining low standard deviations. In contrast, IDM + MOBIL lagged slightly behind in this metric, with an average speed of only 24.8 m/s and a standard deviation nearly three times that of CPO and RECPO. As for the DDPG algorithm, despite undergoing 3000 epochs of training, it seemed unable to adapt to the test environment, showing very poor results in efficiency, comfort, and safety. Overall, in terms of driving efficiency, the performance of RECPO and CPO was better than the built-in autonomous driving algorithm IDM + MOBIL in the Carla simulator. They were faster and more stable than the IDM + MOBIL algorithm, demonstrating better adaptability of RECPO and CPO Specific data are shown in Table 2.

In terms of passenger comfort, the RECPO algorithm maintains an average acceleration below 0.1 m/s^2^ throughout the drive, with a standard deviation of only 0.88 and a jerk mean of −0.098 m/s^3^. This indicates a very stable vehicle speed during high-speed travel, where passengers hardly feel any acceleration due to throttle or brake applications. Although the IDM + MOBIL also maintains an average acceleration under 0.1 m/s^2^, its higher standard deviation of 3.25 and a jerk mean of −0.171 m/s^3^ suggest less stability in acceleration, leading to a more pronounced feeling of bumpiness compared to RECPO. Despite the CPO algorithm exhibiting a higher average acceleration than both RECPO and IDM + MOBIL, its standard deviation is low at 0.85, and its jerk mean is only −0.058 m/s^3^, indicating higher stability in acceleration. Even though there is a stronger sensation of thrust and pitching during acceleration and braking phases, the vibration is less than that of IDM + MOBIL. The average acceleration is 1.21 m/s^2^, which is acceptable for highway driving. Detailed data can be found in Table 3.

In terms of safety, RECPO, CPO, and IDM + MOBIL are at one extreme, with DDPG performing very poorly at the other. DDPG failed to complete any test rounds, either colliding with other vehicles or veering off the road. In contrast, RECPO and CPO, due to their independent cost functions and inclusion of safety distances as a component of these cost functions, performed much better in safety compared to IDM + MOBIL. The training curves and test results show that CPO did not fully converge like RECPO at 3000 epochs, highlighting RECPO’s faster convergence rate.Specific data are shown in Table 4.

It is noteworthy that in our data statistics for lane changes and lane keeping, IDM + MOBIL rarely performs lane changes. In contrast, RECPO initiates a lane change when the distance to the vehicle ahead is approximately $d_{safe}$ + 3.72 m, and CPO does so at about $d_{safe}$ + 2.76 m. Apart from these instances, both systems consistently maintain their lanes.During each testing round, the number of lane changes for both algorithms depends on vehicle density, averaging less than two changes per test.

In general, RECPO and CPO demonstrated higher driving efficiency, better passenger comfort, and safer driving in test experiments, showing excellent adaptability and learning capabilities. Moreover, RECPO has a stronger adaptability and faster convergence rate compared to CPO.

## 7. Conclusions

This paper proposes a decision-making framework for highway autonomous driving that prioritizes driving safety and ensures robust performance. The framework employs a CPO method to construct an RL policy that maximizes rewards while ensuring compliance with safety constraints. We introduced importance sampling techniques, allowing the agent to collect and store data in a Replay buffer during execution, thus supporting the new policy model with data from old policies and effectively preventing catastrophic forgetting. Additionally, we framed the highway autonomous driving decision problem as a CMDP and employed an improved RECPO method for training to optimize the autonomous driving strategy. Finally, by deploying this method in the CARLA simulation environment and comparing it with traditional CPO methods, advanced DDPG-based highway autonomous driving strategies, and the IDM + MOBIL method, the experimental results demonstrate enhancements in the model’s convergence speed, safety, and decision stability offered by our proposed framework. However, our work still requires improvement. Firstly, we have only considered a three-lane highway under normal conditions, but in the real world, highways often feature ramps, multi-lane roads, and varying speed limits by lane, all of which increase the difficulty of autonomous driving and could compromise driving stability. Secondly, we have not considered scenarios where the EV or SV needs to change lanes to an emergency lane in urgent situations. Additionally, the observation space in our study is based on precise acquisition from a simulation space, whereas in the real world, sensor data acquisition is always noisy and may even involve sensor errors. To address these issues, we plan to introduce more complex strategic neural networks (such as transformers) or hierarchical decision frameworks in our future work, enabling the strategy to adapt to more complex and random autonomous driving scenarios and enhance driving stability and safety.

## Figures and Tables

**Table 1:** Experimental parameter settings.

Parameters	Value
***CARLA Simulator***	-
Time step $t_{step}$	0.05 s
Maximum number of training epochs and tiem steps $N_{e}$	5000, 100
Total length of road and simulated road length	10 km, 1 km
Speed limit	[17 m/s, 30 m/s]
Acceleration limit	<3 m/s^2^
Lane width	3.5 m
Number of lanes and vehicles	3, 24
***RECPO & CPO***	-
Discount factor γ and $\lambda_{gae}$	0.9, 0.97
lr for reward and cost	1 × $10^{-3}$→0
Number of hidden layer and hidden layer neuron	2, 128
Distance of safe $d_{safe}$, observation $d_{o}$ and target waypoint $d_{w}$	30 m, 50 m, 2 m
Weight of cost function $k_{1},k_{2},k_{3},k_{4}$	45, 50, 5, 5
Weight of reward function $k_{5},k_{6},k_{7}$	2, 1, 50
Replay Buffer size	20,480
***DDPG***	-
lr for actor and critic	1 × $10^{-4}$→0
Optimizer of actor and critic	Adam
Number of hidden layer	2
Number of hidden layer1 and layer2 neuron	400, 300
Discount factor γ	0.99
Replay Buffer size	1,000,000

**Table 2:** Efficiency simulation performance test results.

Methods	Average Speed	Speed Standard Deviation
IDM + MOBIL	24.81	5.95
RECPO	27.52	0.88
CPO	27.28	1.73
DDPG	44.57	19.41

**Table 3:** Comfortable simulation performance test results.

Methods	Average Acceleration	Acceleration Standard Deviation	Average Jerk
IDM + MOBIL	−0.0321	3.25	−0.171
RECPO	0.084	0.75	−0.098
CPO	0.21	0.85	−0.058
DDPG	3.14	0.98	−0.7

**Table 4:** Safety simulation performance test results.

Methods	Success Rate	Safe Distance Trigger	Average Front Vehicle Distance
IDM + MOBIL	100%	48	17.73
RECPO	100%	0	58.85
CPO	100%	0	48.53
DDPG	4%	1	32.35

## Data Availability

The data presented in this study are available upon request from the corresponding author.
